# Analysis of Roux-en-Y Gastric Bypass and High-Fat Feeding Reveals Hepatic Transcriptome Reprogramming: Ironing out the Details

**DOI:** 10.3390/jcm15020479

**Published:** 2026-01-07

**Authors:** Matthew Stevenson, Munichandra Babu Tirumalasetty, Ankita Srivastava, Qing Miao, Collin Brathwaite, Louis Ragolia

**Affiliations:** 1 Department of Foundations of Medicine, NYU Grossman Long Island School of Medicine, NYU Langone Hospital–Long Island, 101 Mineola Blvd, Ste. 4-004, Mineola, NY 11501, USA; mrstevenson76@gmail.com (M.S.); munichandra.tirumalasetty@nyulangone.org (M.B.T.); ankita.srivastava@nyulangone.org (A.S.); qing.miao@nyulangone.org (Q.M.); collin.brathwaite@nyulangone.org (C.B.); 2 Department of Surgery, NYU Grossman Long Island School of Medicine, NYU Langone Hospital–Long Island, 101 Mineola Blvd, Ste. 4-004, Mineola, NY 11501, USA

**Keywords:** bariatric surgery, nonalcoholic fatty liver disease (NAFLD), iron homeostasis (hepcidin), gastric bypass, high-fat diet

## Abstract

**Highlights:**

- RYGB alters liver genes, reversing obesity-linked transcriptional changes.
- HFD after RYGB triggers liver stress, inflammation, and iron imbalance.
- Diet quality post-RYGB is key to sustaining liver and metabolic benefits.

**Abstract:**

**Background/Objectives:** Roux-en-Y gastric bypass (RYGB) improves obesity-related metabolic disorders, yet post-operative dietary composition critically shapes outcomes. This study explored how RYGB and high-fat diet (HFD) differentially regulate hepatic transcriptional programs. **Methods:** We performed RNA-seq on liver tissues from diet-induced obese C57BL/6 male mice 8 weeks post-RYGB or sham surgery, maintained on chow or HFD. Differentially expressed genes (DEGs) were identified using DESeq2. Gene sets were categorized as RYGB-induced (commonly regulated by surgery across diets), Reversal (RYGB-driven counter-regulation of obesity-induced changes), and HFD-induced (commonly regulated by diet). A subset of RYGB-specific HFD-induced genes was derived by excluding HFD-induced genes from the RYGB Chow vs. RYGB HFD contrast. Pathway enrichment was conducted using STRING. **Results:** RYGB induced 365 DEGs, including pathways related to extracellular remodeling and reduced mitochondrial/antioxidant activity. Among these, 119 Reversal genes countered obesity-associated transcriptional patterns and accounted for ~27% of the RYGB-induced enrichment results. HFD regulated 860 DEGs, highlighting stress responses and translational repression. Lastly, a set of 426 RYGB-specific HFD-induced genes revealed persistent hepatic inflammation, coagulation, and iron dysregulation under HFD despite surgery. **Conclusions:** RYGB induces robust hepatic transcriptomic changes that attenuate obesity-driven dysregulation, including a coordinated reprogramming of iron-handling pathways. However, high dietary fat partially overrides these benefits, promoting inflammatory, metabolic stress, and iron-related stress. Optimizing post-operative diets and carefully managing micronutrient intake, especially iron, may enhance RYGB’s metabolic efficacy and long-term liver health.

## 1. Introduction

Obesity remains a major global health concern [1,2] and is often associated with serious metabolic disorders, sometimes grouped under the broader term ‘metabolic syndrome’ [3], such as type 2 diabetes and non-alcoholic fatty liver disease (NAFLD) [4]. Roux-en-Y gastric bypass (RYGB) is a commonly performed surgical procedure that can induce significant and often rapid weight loss, leading to improvements in metabolic indicators. However, post-operative diet exerts a decisive influence on these outcomes: high-fat (HF) intake can undermine many of the metabolic gains otherwise seen after RYGB, suggesting a close interplay between dietary composition and surgical intervention.

In a recent companion study using the same mouse cohort [5], we demonstrated that while RYGB markedly lowered body weight and improved dyslipidemia, the degree of post-surgical metabolic improvement varied greatly with dietary composition. Although RYGB has been proposed as a potential treatment for NAFLD [6,7,8,9,10,11], our findings revealed that HF feeding post-RYGB constrained many of its metabolic benefits, including incomplete resolution of NAFLD and hepatic fibrosis. HF feeding also sustained elevated fasting glucose levels. Interestingly, RYGB led to systemic iron deficiency under both diets, suggesting that altered iron handling may be intertwined with the very metabolic adaptations that promote weight loss and improved lipid profiles. Given iron’s importance in mitochondrial function and redox balance, these findings suggest that while RYGB reshapes metabolic pathways, dietary composition ultimately dictates the extent and durability of these effects.

By expanding the analysis here to include global hepatic gene expression, we aim to uncover the molecular pathways that underlie the interplay between RYGB and dietary fat intake. Because the liver is a central hub governing lipid and glucose metabolism, inflammation, and micronutrient handling, we performed a transcriptome-wide investigation of liver tissue in mice subjected to either RYGB or sham surgery, each group fed either chow or a high-fat diet (HFD). Our goal is to identify molecular signatures and pathways governed by surgical intervention, by diet, or by their combination. Given that RYGB mice exhibited increased fecal lipid loss under HF feeding, transcriptomic changes in lipid metabolism may reveal adaptive hepatic responses to altered nutrient absorption. Similarly, persistent hepatic inflammation and fibrosis in RYGB + HFD mice suggest transcriptional signatures that may explain why HF feeding attenuates RYGB’s hepatoprotective effects. Finally, given that RYGB-induced iron deficiency was present regardless of diet, we seek to determine whether transcriptional reprogramming of hepatic iron-handling pathways contributes to this phenomenon beyond malabsorption alone. Through this, we aim to shed light on how specific dietary habits can either reinforce or limit RYGB’s potential to improve metabolic health.

## 2. Methods

### 2.1. Experimental Design, Sample Collection and Preparation

**Figure 1:**
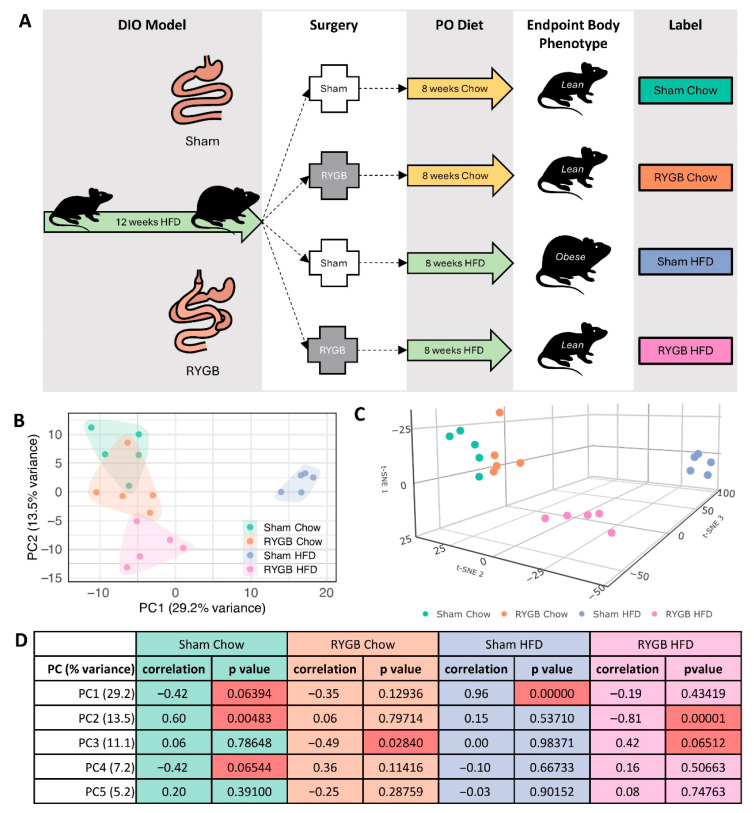
Distinct Transcriptomic Landscapes Shaped by Surgical Intervention and Dietary Composition. (**A**) Male C57BL/6 mice were fed a high-fat diet (HFD) for 12 weeks to induce obesity before undergoing RYGB or Sham surgery. Post-surgery, mice were maintained on Chow or HFD for 8 weeks, resulting in four groups: Sham Chow, RYGB Chow, Sham HFD, and RYGB HFD. Phenotypes and group color codes are indicated here and are maintained throughout this study. (**B**) PCA shows clustering by experimental group along PC1 and PC2, with convex hulls highlighting group separation. Axes indicate the variance explained by each principal component. (**C**) t-SNE visualization shows distinct clustering of transcriptional profiles by group in three-dimensional space. (**D**) Pearson correlation coefficients and *p*-values for the top five PCs were calculated against experimental groups, with significant and near-significant correlations highlighted in red. Variance contributions for each PC are shown in parentheses alongside each PC.

Diet-induced obese male C57BL/6 mice underwent either RYGB or sham surgery and subsequently maintained on either a chow or HFD for 8 weeks post-operatively (Figure 1A). Diet-induced obese male C57BL/6J mice (average preoperative weight 41.49 ± 4.45 g), purchased from The Jackson Laboratory (Farmington, CT, USA), underwent either RYGB or sham surgery and were anesthetized with isoflurane during surgery, and subsequently maintained on either a chow or HFD for 8 weeks post-operatively (Figure 1A). Full details regarding the surgical procedures, post-operative care, and dietary regimens are provided in the original publication [5]. At the conclusion of this study, mice were euthanized via cervical dislocation, and liver tissues were snap frozen to preserve RNA integrity for subsequent analyses. The experimental unit was the individual mouse, with one liver sample collected per animal. For RNA-seq, five liver samples per group were analyzed (*n* = 5 per group; total *n* = 20 across four groups: Sham Chow, Sham HFD, RYGB Chow, and RYGB HFD), with samples randomly selected from the full cohort described in the previously published study. Animals were not allocated to experimental groups as part of the present RNA-seq study; rather, liver samples were randomly selected within each group from the parent cohort. No additional steps were taken in this secondary RNA-seq analysis to control potential confounders (e.g., processing order or animal/cage location) beyond the standardized conditions described for the parent study. No a priori sample size calculation was performed for this secondary transcriptomic analysis; the subset size was determined by feasibility and sequencing cost while maintaining balanced group sizes. As no a priori sample size calculation was performed, no primary outcome measure was used to determine sample size. No animal-level or sample-level inclusion or exclusion criteria were applied beyond group assignment, and no animals or liver samples were excluded from the RNA-seq analysis. All downstream analyses were performed using *n* = 5 per group. Blinding was not performed for RNA-seq sample selection, processing, or data analysis; blinding during animal allocation and conduct in the parent study was not reported. These liver tissues, obtained from the previously published study were used for total RNA extraction with Trizol reagent (Ambion, Life Technologies, Carlsbad, CA, USA), followed by purification and concentration using RNeasy MinElute Cleanup Kit (Qiagen, Hilden, Germany; #74204). RNA purity was assessed by 260/280 and 260/230 ratios and samples were sent for sequencing. Animal experiments were approved by the NYU Grossman Long Island School of Medicine IACUC and conducted in accordance with the National Institutes of Health guide for the care and use of Laboratory animals (NIH Publications No. 8023, revised 1978) and reported in accordance with ARRIVE guidelines.

### 2.2. RNA Sequencing and Library Preparation

The extracted RNA from the liver tissue was evaluated for both quantity and integrity. RNA integrity numbers (RIN) were calculated to guarantee high-quality RNA for sequencing. Following its separation from the total RNA by means of magnetic beads coupled to poly-T oligos, the mRNA was then broken into tiny fragments by divalent cations at high temperatures.

Reverse transcriptase and random hexamer primers were used for the first strand of cDNA synthesis, and DNA Polymerase I and RNase H were used for the second strand. dUTP was added during second-strand synthesis to maintain strand specificity in directed libraries. Following that, the cDNA was subjected to adapter ligation, A-tailing, and end repair. To separate cDNA fragments that were most suited for sequencing, size selection was carried out.

An Agilent Bioanalyzer and a Qubit Fluorometer were used to assess the produced libraries’ quality. Real-time PCR was used to quantify the libraries, and the Bioanalyzer was used to confirm their size distribution. Libraries were pooled and sequenced on an Illumina platform using a paired-end 150 bp (PE150) technique based on their effective concentration and total data [12]. All these procedures were carried out by Novogene in Sacramento, CA, USA.

### 2.3. Data Processing

Initial processing of raw sequencing reads involved eliminating adapters, poly-N sequences, and low-quality fragments using Trimmomatic v0.36 with default settings [13]. Subsequently, the read quality of each sample was evaluated using the FastQC program with standard. The pre-processed reads were aligned to mouse reference genome, mm10 through HISAT2 v.2.1.0 program using the splice-mapping approach with default setting [14]. Furthermore, FeatureCounts v1.5.0-p3 was used to quantify the number of reads for each gene [15]. Using FPKM and read count as the main metrics, gene expression levels were evaluated using FPKM (Fragments Per Kilobase of Transcript Sequence per Million Base Pairs), which was computed using the length of the gene and the number of reads mapped to it.

To ensure high-quality data, raw read counts were visualized (Supplementary Figure S1A) and subjected to a count-per-million (CPM) filter of 2 in at least three samples. This threshold was determined based on signal-to-noise ratio (SNR) assessments and log2 CPM distribution analyses, which identified transition ranges between noise and true signal at 0.59–5.1 CPM and 1.82–3.92 CPM, with mean values around 2.84 and 2.87 CPM (Supplementary Figure S1B–D). The density plot of expression levels (Supplementary Figure S1E) revealed a consistent distribution following noise removal, reflecting balanced sensitivity and specificity. A CPM threshold of 2 aligns with general conventions (1–2 CPM) while ensuring detectable gene expression across multiple samples and mitigating noisy signals.

### 2.4. Differential Expression Analysis

Differential expression (DE) analysis was conducted using DESeq2 [16] in RStudio (Version 2024.12.0+467) [17]. We focused on four pairwise contrasts to address potential confounding variables, particularly body weight differences, and provide a clear view of transcriptional changes. Raw count data were normalized using DESeq2’s median-of-ratios method, applying a design formula of ~Group. To improve interpretability and stability of effect size estimates, lfcShrink [18] was applied, further refining log2-fold change values for downstream analyses. Group labels were dynamically re-leveled for each comparison, allowing for robust identification of transcriptional shifts across the following key contrasts:

- Sham Chow vs. RYGB Chow;
- Sham HFD vs. RYGB HFD;
- RYGB Chow vs. RYGB HFD;
- Sham Chow vs. Sham HFD.

From the outset, body weight discrepancies between groups, previously characterized in detail [5], posed a significant challenge. Sham HFD mice exhibited notably higher body weights than all other groups, introducing a confounding variable that biased transcriptional patterns (Supplementary Figure S2A). Additionally, contrasts involving the Sham HFD condition disproportionately preserved significant genes after multiple-testing correction. For instance, in the Sham HFD vs. RYGB HFD comparison, 59.2% of the upregulated genes and 61.8% of downregulated genes remained significant post-correction; by contrast, only 9.7% and 3.9% were retained in the Sham Chow vs. RYGB Chow comparison (Supplementary Figure S2B,C). These outcomes highlighted the risks of stringent FDR-based thresholds, where biologically validated changes (e.g., *Hamp*) were sometimes excluded despite showing large fold changes and corroboration by independent experiments [5].

Given these issues, we deemed factorial designs unsuitable. Residual confounding, particularly among comparisons incorporating the Sham HFD group, could not be adequately controlled within a single factorial model. Accordingly, we adopted a paired-comparison approach validated by biological replication and permutation testing.

### 2.5. Identification of Commonly Regulated Genes

To determine which genes were consistently altered across various experimental conditions, we first inspected the results of four pairwise DE comparisons (Sham Chow vs. RYGB Chow, Sham HFD vs. RYGB HFD, RYGB Chow vs. RYGB HFD, and Sham Chow vs. Sham HFD). Because body weight discrepancies precluded a single factorial framework, each contrast was treated independently, prioritizing unadjusted *p*-values (<0.05) to capture biologically meaningful alterations that might be missed by more conservative statistical thresholds.

In identifying RYGB-induced effects, we specifically looked for genes that reached significance in both Sham Chow vs. RYGB Chow and Sham HFD vs. RYGB HFD and that showed the same direction of change in both comparisons (Supplementary Figure S3A). This permitted the identification of transcripts whose regulation was robust under surgical intervention, irrespective of diet.

Next, we aimed to identify genes that not only responded to RYGB but also opposed the “obese” transcriptomic state typically induced by HF feeding. By overlapping the RYGB-induced genes with those showing differential expression in Sham Chow vs. Sham HFD, we derived a set of “Reversal” genes. These exhibited significant RYGB-induced changes in the opposite direction of the obesity signature (Supplementary Figure S3B). We interpret this subset as potentially central to countering obesity-related perturbations.

Likewise, we examined diet-induced shifts independently of surgery. Genes that were significantly and consistently altered (i.e., regulated in the same direction) in both Sham Chow vs. Sham HFD and RYGB Chow vs. RYGB HFD were merged to pinpoint transcripts broadly affected by HFD (Supplementary Figure S3C). By requiring significance in both diet-based comparisons, we identified genes that represent consistent HFD effects across both surgical and non-surgical states, as well as across distinct body phenotypes (lean vs. obese and lean vs. lean). We term these “HFD-induced,” as they capture gene-expression patterns strongly linked to HF feeding, irrespective of RYGB or body weight.

Taken together, these overlapping gene sets (RYGB-induced, Reversal, and HFD-induced) formed the basis of the subsequent pathway enrichment analyses. By filtering transcripts that met unadjusted *p* < 0.05 across relevant contrasts, we could emphasize biological reproducibility rather than potentially restrictive multiple-testing criteria. Follow-up validation using permutation analysis (outlined below) confirmed that the observed overlapping gene signatures did not arise simply by chance but revealed coherent transcriptomic programs involving surgery, diet, or both.

### 2.6. Permutation Analysis for Validation

Permutation analysis was used to validate the biological significance of commonly regulated genes and assess how likely significant overlaps might occur by random chance. For RYGB-induced effects, surgery labels (Sham vs. RYGB) were randomly shuffled within each diet condition (Chow or HFD) in 10,000 iterations, generating null distributions that preserved study design. At each iteration, we repeated the DE analysis (*p* < 0.05) and recorded the overlap between permuted Chow and HFD contrasts.

To ensure that the HFD-induced gene set was similarly robust, we performed an analogous analysis in which diet labels (Chow vs. HFD) were permuted within each surgical group (Sham or RYGB). Again, 10,000 iterations were run, and the overlap of significantly altered genes in the permuted datasets was recorded.

This empirical approach circumvented the limitations of conventional FDR corrections in the presence of confounding variables [19,20,21,22,23].

$$P=\frac{Number\;of\;Permuations\;with\;Overlap\;Observed+1}{Total\;Permutations+1}$$

### 2.7. Refinement of RYGB-Specific HFD Effects

To isolate transcripts uniquely governed by diet within the RYGB condition, we removed genes previously identified as HFD-induced from the RYGB Chow vs. RYGB HFD results (Supplementary Figure S3D). The remaining genes were then filtered using an adjusted *p*-value (<0.05). Unlike the unadjusted approach taken for commonly regulated gene sets, this final filter was designed to capture genes specifically echoing the interaction of RYGB and diet, rather than broad HFD-driven changes.

### 2.8. Pathway Enrichment Analysis

Pathway enrichment was performed on the refined gene lists, namely RYGB-Induced, Reversal, HFD-Induced, and RYGB-Specific HFD-Induced, each derived from iterative filtering strategies to capture distinct biological contexts. Analyses used STRING (Search Tool for the Retrieval of Interacting Genes/Proteins) version 12.0 to identify functional associations from Gene Ontology (GO), KEGG, Reactome, and WikiPathways [1,24,25,26,27,28,29,30,31,32,33,34,35,36,37]. A minimum interaction score of 0.4 (medium confidence) was used to filter protein–protein interactions, and all evidence sources (e.g., experimental data, computational predictions, text mining) were included. A custom background restricted to expressed genes in this dataset was employed to enhance specificity when identifying functional terms.

To reduce overlapping or redundant pathway annotations, a two-step clustering process was applied: first, gene-set clustering based on Jaccard distances of shared membership was used to group similar biological terms; second, text-based clustering merged near-duplicate annotations (e.g., GO vs. KEGG) that effectively labeled the same pathway. The final enrichment results were visualized in lollipop-style plots showing the top ten pathways per list (ranked by signal, a combined score derived from FDR and strength).

### 2.9. PubMed Gene Association Analysis

To evaluate the PubMed literature association of HFD-induced genes, a keyword-based search was performed using programmatic queries in R. The “HFD” category included keywords such as “high-fat diet,” “HFD,” “high-fat feeding,” and “fat-rich diet,” while the “obesity” category included terms like “obesity,” “adiposity,” “weight gain,” and “body mass index.” The total number of PubMed hits for each gene under both categories was retrieved and compared.

### 2.10. Heatmap Generation

Heatmaps generated using pheatmap illustrate transcriptional shifts among the refined gene sets derived from our pairwise comparisons. For the commonly regulated gene sets, an iterative percentile approach was employed to select a consistent number of top-ranked genes (e.g., Top 25) for each condition based on *p*-value or log2 fold change (log2FC), ensuring uniform representation of highly responsive transcripts. Rlog-transformed expression values were row-scaled. Hierarchical clustering arranged genes by expression profile, and columns were sorted based on the ordering used in our experimental design graphic (Figure 1A) and previous publication [5], maintaining consistency with this study’s established framework. This approach offers an intuitive depiction of the principal transcriptional modulations tied to diet (Chow vs. HFD), surgery (Sham vs. RYGB), as well as differences observed across these conditions.

For RYGB-Specific HFD Effects, heatmaps were generated using the top 25 DEGs by the adjusted *p*-value and log2FC. This selection highlights the most prominent transcriptional differences confined to RYGB’s response to HFD.

## 3. Data Availability

Raw sequencing data (FASTQ files) and gene-level count matrices have been deposited in the NCBI Gene Expression Omnibus (GEO) under accession number GSE303852 at the following link:

https://urldefense.com/v3/__https://www.ncbi.nlm.nih.gov/geo/query/acc.cgi?acc=GSE303852__;!!MXfaZl3l!Y_krM93hJyi58Hf2gD--A4FRqZGV1DIv1-tzsb6HO5PM5fBicmwbZyDShI2LFB2pAHoSOB9fRS9UZeE8OBE6Xenwbw$ (accessed on 19 August 2025)

The token for access is: wzwfgmgqzvwpzer.

Curated gene lists derived from our analytical framework, including those used for identifying RYGB-induced, Reversal, HFD-induced, and RYGB-specific transcriptomic changes, are available from the corresponding author upon reasonable request.

## 4. Results

### 4.1. Distinct Transcriptomic Landscapes Shaped by Surgical Intervention and Dietary Composition

We analyzed RNA-seq profiles from liver samples collected 8 weeks post-surgery (RYGB or Sham) under chow or HFD (Figure 1A).

Following the removal of lowly expressed genes using a CPM threshold of 2 in at least three samples, 12,289 genes remained for further analyses. Exploratory methods, such as principal component analysis (PCA) and t-distributed stochastic neighbor embedding (t-SNE), revealed distinct group separations driven by both surgery and diet (Figure 1B,C). Lean groups clustered distinctly from obese groups along PC2, demonstrating a robust body phenotype imprint on the hepatic transcriptome. Within the lean groups, clear separation was observed among Sham Chow, RYGB Chow, and RYGB HFD in the 3D t-SNE plot. This separation, arising from contributions across all three t-SNE dimensions, emphasizes the unique transcriptional profiles associated with both surgical intervention and dietary composition, independent of body phenotype. Additionally, distinct clustering within diet groups further emphasized how dietary composition profoundly shapes pathways linked to lipid metabolism and inflammation.

To further explore these patterns, we calculated correlations between the top five principal components (PCs) and experimental groups (Figure 1D). Significant correlations were observed for specific PCs, while others showed trends suggestive of potential associations. For example, PC1 exhibited a strong positive correlation with Sham HFD (r = 0.96, *p* < 0.00001), and a trend toward a negative correlation with Sham Chow (r = −0.42, *p* = 0.064). PC2 showed a significant positive correlation with Sham Chow (r = 0.60, *p* = 0.0048) and a strong negative correlation with RYGB HFD (r = −0.81, *p* < 0.00001). In contrast, PC3 showed a near-significant trend for a negative correlation with RYGB Chow (r = −0.49, *p* = 0.028) and a trend toward a positive correlation with RYGB HFD (r = 0.42, *p* = 0.065). Other PCs showed weaker or non-significant correlations. These relationships highlight the distinct contributions of diet and body phenotype to the overall variance in gene expression, underscoring the complementary roles of PCA and t-SNE in delineating transcriptional changes across experimental conditions.

These findings dovetail with observations from the previous study of these mice [5], in which RYGB improved NAFLD-related features under chow and, to a lesser extent, under HFD. RYGB Chow mice showed marked resolution of steatosis and inflammation, whereas RYGB HFD exhibited milder improvements. Elevated fibrosis scores and lobular inflammation persisted in the RYGB HFD group, suggesting some ongoing hepatic stress, despite metabolic improvements attributable to the surgical procedure. Such exploratory analyses confirmed that the dataset was well-suited for in-depth differential expression and pathway-level investigations.

### 4.2. RYGB-Driven Hepatic Transcriptomic Remodeling: Enhanced Extracellular/Adipogenesis Pathways and Reduced Metabolic Activity

Our RNA-seq analysis identified 365 genes (136 upregulated, 229 downregulated; Supplementary Figure S4A) that were significantly altered by RYGB when compared to sham controls under both dietary conditions.

**Figure 2:**
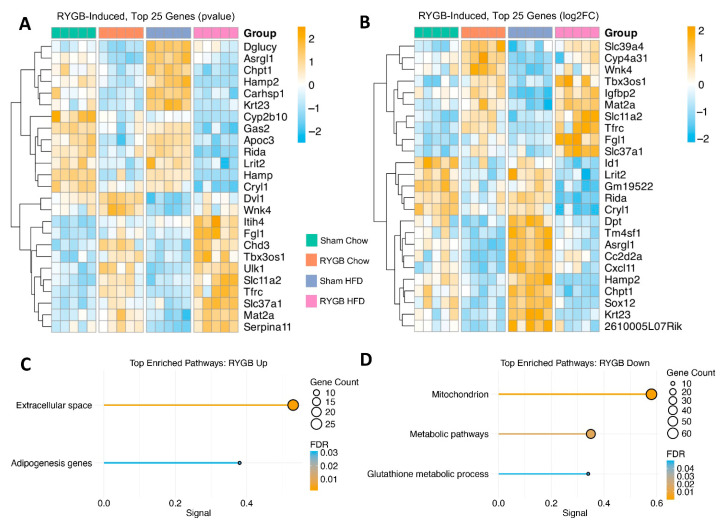
RYGB-Driven Hepatic Transcriptomic Remodeling: Enhanced Extracellular/Adipogenesis Pathways and Reduced Metabolic Activity. Heatmap illustrating the top 25 RYGB-induced genes, selected using an iterative percentile approach to ensure consistent representation of highly responsive transcripts based on (**A**) *p*-value and (**B**) log2FC. Scaled rlog-transformed expression values are shown for each experimental group, with hierarchical clustering of genes by expression profile. Pathway enrichment analysis reveals (**C**) upregulated pathways associated with extracellular remodeling and adipogenesis and (**D**) downregulated pathways involving mitochondrial function, general metabolism, and glutathione-related antioxidant processes.

From this larger pool, subsets of 25 genes were prioritized based on robust *p*-value (Figure 2A) and fold change (Figure 2B, see Methods), capturing the most pronounced surgery-driven transcriptional shifts under chow or HFD. Using comparisons of the observed overlap with the null distribution, permutation analysis demonstrated that the actual overlap of the RYGB-induced genes significantly exceeded permutation-based expectations (*p* < 0.0002), underscoring the statistical and biological stability of these shared gene sets (Supplementary Figure S5A).

Among the RYGB-Induced genes, two pathways emerged as significantly enriched in the upregulated set (Figure 2C). One, “Extracellular space” (FDR = 0.0014, Signal = 0.53), included secretory and matrix-associated factors such as *Igfbp2*, *Inhba*, *Cd9*, and *Lsr* as well as several iron metabolism genes such as *Tfrc*, *Fgl1*, *Hp*, and *Pzp*, suggesting RYGB-driven changes in tissue architecture, intercellular signaling, or possibly iron homeostasis. A second upregulated pathway, “Adipogenesis genes” (FDR = 0.0310, Signal = 0.38), contained *Socs3*, *Trib3*, and *Epas1* (*Hif2α*), all of which have been implicated in both adipocyte differentiation and hepatic adaptation.

In contrast, three pathways were downregulated after RYGB, primarily linked to mitochondrial and metabolic functions (Figure 2D). The “Mitochondrion” pathway (FDR = 0.000057, Signal = 0.58) encompassed dozens of genes including *Acad9*, *Sod2*, *Rida*, *Dglucy*, and *Prkaca* that collectively point to a shift in mitochondrial activity or oxidative stress regulation. Another broad metabolism term, “Metabolic pathways” (FDR = 0.0144, Signal = 0.35), included *Cryl1*, *Asrgl1*, *Chpt1*, *Dglucy*, and *Cyp2b10*, highlighting a possible drop in metabolic flux and impaired substrate utilization. Additionally, the “Glutathione metabolic process” (FDR = 0.0497, Signal = 0.34) included *Gstk1*, *Gstz1*, and *Park7*, suggesting that hepatic antioxidant demands might lessen in response to RYGB. Of note, several of these downregulated genes ranked among the top 25 DEGs by both fold change and *p*-value (e.g., *Rida*, *Chpt1*, *Cryl1*, and Asrgl1), whereas *Dglucy* and *Cyp2b10* were top 25 DEGs by *p*-value alone, underscoring their potential importance in RYGB-induced metabolic remodeling.

Within the downregulated genes, key regulators of lipid metabolism were also noted, including *ApoC3*, *ApoC1*, *ApoA2*, *ApoA5*, and *Angptl3*. These genes are involved in triglyceride-rich lipoprotein production, clearance, and lipolysis regulation. While pathways specifically linked to lipid metabolism or lipolysis did not show significant enrichment, their consistent downregulation suggests a coordinated shift in lipid metabolism, contributing to the triglyceride-lowering effects observed after RYGB [38].

Overall, these transcriptomic shifts resonate with phenotypic changes documented in these RYGB mice, including reduced plasma TG and LDL levels and consistent modulation of iron-related genes like *Tfrc*, *Slc11a2* (*Dmt1*), *Hamp*, aligning with known iron deficiency patterns observed after RYGB [39] and in the previous study of these mice [5]. Moreover, although Sham Chow alone was sufficient to normalize many NAFLD features, the additional enrichment of mitochondrial and “adipogenesis”-related transcripts in RYGB mice suggests an extra layer of hepatic adaptation under challenging dietary conditions.

### 4.3. Counteracting Obesity-Linked Dysregulation: RYGB-Mediated Reversal of DIO-Associated Gene Signatures

After identifying 365 RYGB-induced genes, we next focused on a subset that specifically countered classical diet-induced obesity (DIO) signatures. This approach yielded 119 (43 upregulated, 76 downregulated; Supplementary Figure S4B) “Reversal” genes, i.e., those regulated in the opposite direction compared to the Sham Chow vs. Sham HFD contrast. By singling out genes that RYGB appears to reverse, we gain clearer insights into the surgery’s unique capacity to override or negate obesity-associated dysregulation, beyond what diet alone can achieve.

**Figure 3:**
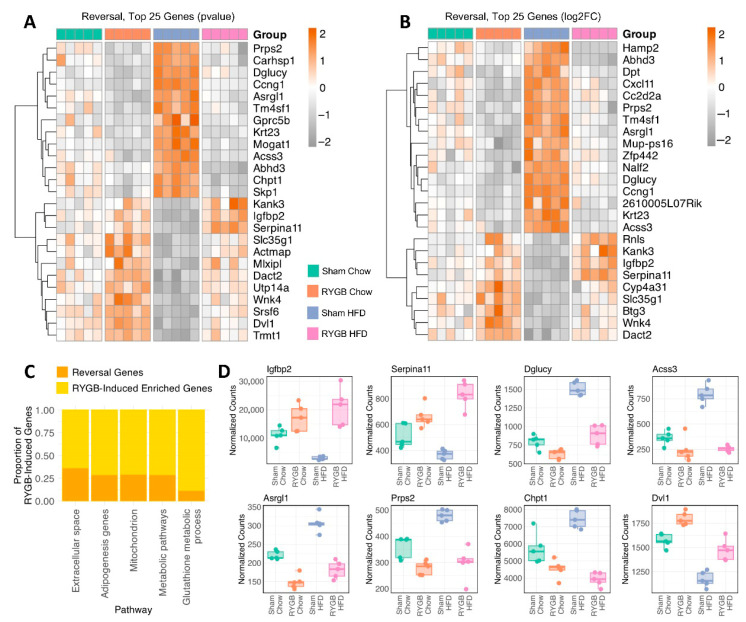
Counteracting Obesity-Linked Dysregulation: RYGB-Mediated Reversal of DIO-Associated Gene Signatures. Heatmap illustrating the top 25 Reversal genes, selected using an iterative percentile approach to ensure consistent representation of highly responsive transcripts based on (**A**) *p*-value and (**B**) log2FC. Scaled rlog-transformed expression values are shown for each experimental group, with hierarchical clustering of genes by expression profile. (**C**) Bar chart depicting the proportion of Reversal genes contributing to enriched pathways highlights key processes, including extracellular remodeling, adipogenesis, and metabolic functions. (**D**) Boxplots of normalized expression counts for Igfbp2, Serpina11, Dglucy, Acss3, Asrgl1, Prps2, Chpt1, and Dvl1. Reversal genes demonstrating consistent reversal of obesity-driven transcriptional changes that were in the Reversal top 25 heatmaps and present in RYGB-induced enriched pathways.

Although we did not observe direct pathway enrichments in this Reversal subset alone (likely due to its moderate size) we contextualized these genes within the broader RYGB-Induced transcriptome. “Top 25” Reversal heatmaps were created based on robust *p*-values (Figure 3A) and fold changes (Figure 3B) across all three comparisons, demonstrating expression patterns distinct from straightforward calorie restriction, i.e., Sham Chow.

To gauge the functional significance of the Reversal subset, we quantified its overlap within each RYGB-Induced pathway. These 119 genes accounted for approximately 27% of the five pathways identified in the RYGB-Induced transcriptional changes (Figure 3C). Eight genes (Figure 3D) appeared in one or both of the “Top 25” Reversal heatmaps, with *Igfbp2*, *Serpina11*, *Dglucy*, *Acss3*, *Asrgl1*, and *Prps2* present in both, and were part of the 27% mentioned above, highlighting their consistent and substantial transcriptional changes. Notably, *Igfbp2* and *Serpina11* were associated with pathways such as ‘Extracellular space’ in the broader RYGB-Induced analysis, suggesting potential roles in reshaping extracellular or signaling environments to reverse obesity-linked liver perturbations.

Lastly, we compared gene abundance with PubMed “hits” metrics for the individual categories “obesity,” “liver,” “RYGB,” “NAFLD,” and “Iron” (Supplementary Figure S6A–E) and then examined an aggregated metric across all categories (Supplementary Figure S6F). Including abundance as an axis allows us to identify genes that are not only potentially novel but also highly expressed, making them more accessible for experimental study and manipulation. This analysis helps distinguish heavily studied genes from more obscure regulators. These Reversal transcripts represent high-priority candidates for understanding how RYGB mechanistically counteracts obesity-associated liver dysregulation.

### 4.4. High-Fat Diet-Driven Hepatic Transcriptomic Reprogramming: Amplified Stress Response and Repressed Protein Synthesis

**Figure 4:**
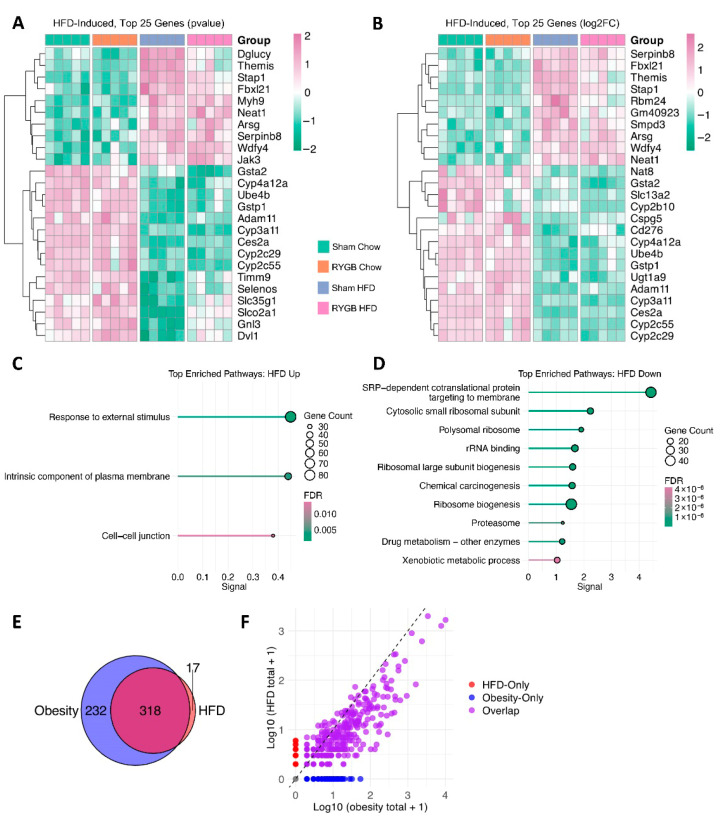
High-Fat Diet–Driven Hepatic Transcriptomic Reprogramming: Amplified Stress Response and Repressed Protein Synthesis. Heatmap illustrating the top 25 HFD-induced genes, selected using an iterative percentile approach to ensure consistent representation of highly responsive transcripts based on (**A**) *p*-value and (**B**) log2FC. Scaled rlog-transformed expression values are shown for each experimental group, with hierarchical clustering of genes by expression profile. Pathway enrichment analysis highlights (**C**) upregulated processes such as response to external stimuli, plasma membrane components, and cell–cell junctions, while (**D**) downregulated pathways include those related to translational suppression, proteasome activity, and xenobiotic metabolism. (**E**) Venn diagram shows the overlap between HFD-induced genes and PubMed associations for “obesity” and “HFD,” emphasizing shared and distinct representation. (**F**) Scatterplot of log10 PubMed hits for genes associated with “HFD” and “obesity” illustrates shared, HFD-specific, and obesity-specific genes.

To disentangle the influence of HF feeding from that of surgery, we applied a strategy like the one used in identifying RYGB-mediated effects. Specifically, we merged significant genes from Sham Chow vs. Sham HFD and RYGB Chow vs. RYGB HFD, defining a set of 860 HFD-Induced genes (403 upregulated, 457 downregulated; Supplementary Figure S4C). This step isolated genes reliably regulated by HF intake regardless of surgical alteration. As before, permutation analysis demonstrated that the actual overlap of the HFD-induced genes significantly exceeded permutation-based expectations (*p* < 0.0001, Supplementary Figure S5B) and highlights the statistical and biological rigor of these shared gene sets. As with previous analyses, subsets of 25 genes were prioritized based on robust *p*-values (Figure 4A) and fold changes (Figure 4B), capturing the most pronounced HFD-driven transcriptional shifts under sham or RYGB.

Enrichment analyses identified 3 upregulated and 34 downregulated pathways in our dataset. In particular, pathways linked to cellular structure or environmental stress responses were strongly induced by HFD (Figure 4C). For instance, “Response to external stimulus” (FDR = 0.0015, Signal = 0.45) and “Intrinsic component of plasma membrane” (FDR = 0.0043, Signal = 0.44) reflect a potent transcriptional activation of cell-surface remodeling and stress-response networks. Despite their similar enrichment signals, these pathways differ in the magnitude of gene-level changes: “Response to external stimulus” includes four of our Top 25 upregulated genes (*Wdfy4*, *Stap1*, *Fbxl21*, and *Jak3*), suggesting a more focused immune or stress-related activation, whereas “Intrinsic component of plasma membrane” contributes fewer high-magnitude shifts spread across a larger set of moderate changes.

Conversely, HFD significantly downregulated pathways associated with translation, ribosome assembly, and protein synthesis (Figure 4D). Among the top hits, multiple pathways; “SRP-dependent cotranslational protein targeting to membrane,” “Polysomal ribosome,” “Ribosomal large subunit biogenesis,” “Ribosome assembly,” and “Ribosome biogenesis”, showed strong signals and very low FDR values, collectively indicating broad repression of ribosomal function and translational machinery. Beyond translation, other suppressed terms included “Proteasome” and “Xenobiotic metabolic process,” suggesting that HFD can dampen both protein turnover and hepatic compound handling. These HFD-Induced gene-level alterations correlate with phenotypic outcomes we observed under HF feeding: persistent lobular inflammation, elevated liver glycogen, hyperglycemia, dyslipidemia, and NAFLD [5].

Beyond single-pathway enrichment, a few downregulated genes that ranked highly by both fold change and *p*-value appeared across multiple annotations. For instance, *Cyp2c29*, *Cyp3a11*, *Gsta2*, and others each mapped to at least four functional categories, including “Chemical carcinogenesis,” “Drug metabolism—other enzymes,” and “Xenobiotic metabolic process.” This highlights a core cluster of detoxification enzymes that are strongly repressed under HF feeding, suggesting a broad hepatic vulnerability to xenobiotic and oxidative stress. Additionally, although ‘Eicosanoid metabolism via cytochrome P450 monooxygenases’ did not place in the top 10 by signal, 60% of its observed genes (3 out of 5, for instance) are in the top 25 downregulated heatmaps, suggesting a high-intensity shift within this narrower function, one that might be overlooked if we only relied on broad or high-signal categories.

Lastly, to explore potential misattribution of HFD-induced genes to obesity in the literature, we conducted an exploratory analysis of PubMed associations for these genes under “HFD” and “obesity” contexts. As expected, there was significant overlap between the two categories, with 318 shared genes, 232 exclusive to obesity, 17 exclusive to HFD (Figure 4E) and 292 with no hits within either category. This pronounced obesity-exclusive bias aligns with the broader pattern observed across the shared genes, which also exhibited a strong weighting toward obesity-related publications (Figure 4F).

### 4.5. Residual High-Fat Burden in RYGB: Coagulatory Strain, Inflammatory Pressure, and Impaired Detoxification

**Figure 5:**
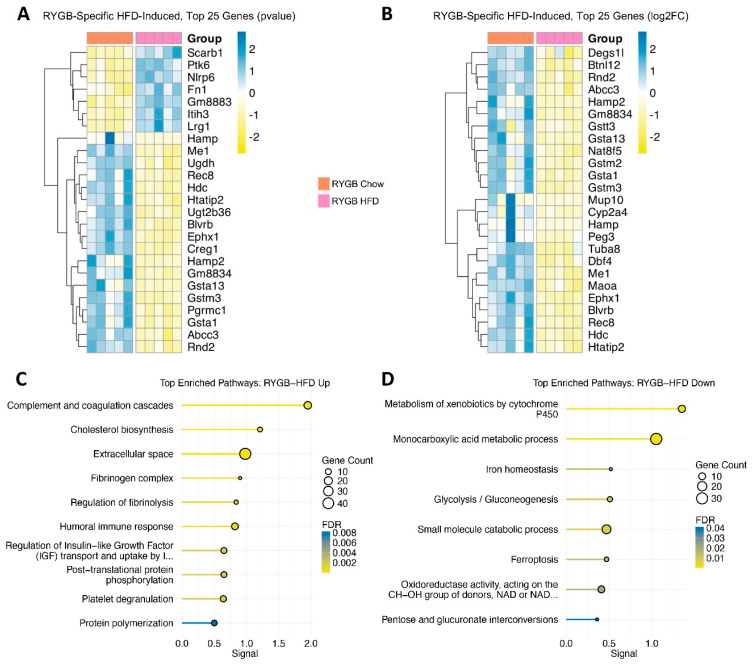
Residual High-Fat Burden in RYGB: Coagulatory Strain, Inflammatory Pressure, and Impaired Detoxification. Heatmap displaying the top 25 RYGB-specific HFD-induced genes ranked by (**A**) *p*-value and (**B**) log2FC. Scaled rlog-transformed expression values are shown for each experimental group, with hierarchical clustering of genes by expression profile. Pathway enrichment analysis highlights (**C**) upregulated pathways related to complement and coagulation cascades, cholesterol biosynthesis, and extracellular space, as well as (**D**) downregulated pathways involving xenobiotic metabolism, glycolysis/gluconeogenesis, and iron homeostasis.

To isolate those diet-driven changes that manifest exclusively in RYGB mice—i.e., an additional layer beyond the ‘core’ HFD-induced effects—we excluded genes commonly regulated by HF feeding (HFD-Induced) from the RYGB Chow vs. RYGB HFD comparison, yielding a final set of 426 genes (229 upregulated, 197 downregulated; Supplementary Figure S4D). This resulting subset highlights how a HFD further reconfigures the hepatic transcriptome when coupled with RYGB-induced anatomical and physiological remodeling. As with previous gene sets, subsets of 25 genes were prioritized based on robust *p*-values (Figure 5A) and fold changes (Figure 5B), capturing the most pronounced HFD-driven transcriptional shifts under RYGB.

In total, 15 upregulated pathways were identified in this analysis, including “Complement and coagulation cascades” (FDR = 1.89 × 10^−10^, signal = 1.95) and “Cholesterol biosynthesis” (FDR = 1.85 × 10^−5^, signal = 1.21), suggesting that HF feeding imposes additional demands on lipid processing and hemostasis even in the post-RYGB state (Figure 5C). Genes such as *Fga*, *Fgb*, and *Fgg* (coding for fibrinogen subunits), along with complement factors like *C3*, *C8a*, and *Cd46*, rose, indicating potential hepatic inflammation or coagulatory strain. Notably, the pathway “Regulation of Fibrinolysis” (FDR = 6.89 × 10^−4^, signal = 0.84) exhibited strength values nearly identical to those of “Fibrinogen Complex,” with both displaying similar FDR and signal scores. This striking similarity may suggest a counterbalancing mechanism, wherein RYGB-induced fibrinolysis (*Plg*, *Klkb1*, *F11*) acts to mitigate fibrosis progression prompted by HFD-driven fibrinogen elevation. This aligns with histological findings showing elevated lobular inflammation and fibrosis in RYGB HFD mice compared to RYGB Chow while still showing an overall reduction in these factors relative to Sham HFD [5].

Conversely, 11 downregulated pathways were identified, including “Metabolism of xenobiotics by cytochrome P450” (FDR = 1.28 × 10^−6^, signal = 1.35) and “Monocarboxylic acid metabolic process” (FDR = 1.89 × 10^−7^, signal = 1.05), pointing toward impaired detoxification and specific intermediary metabolic processes under HFD (Figure 5D). This diminished reliance on oxidative processes may partly reflect our previous finding that these RYGB-HFD mice excrete significantly more lipids in their feces than RYGB Chow mice (38191966), potentially lowering the hepatic influx of fatty acids and thus the need for robust β-oxidation. Reduced expression of iron-handling genes (*Hamp*, *Hamp2*, *Ftl1*, *Fth1*) highlights the likelihood of diet-dependent regulation of hepatic iron metabolism, a notable concern post-RYGB where conditions of anemia often arise. Several glycolytic or gluconeogenic genes, including *Pck2*, *Gpi1*, and *Pfkm*, were also diminished, possibly contributing to the heightened glycogen stores and hyperglycemia persisting in RYGB HFD relative to RYGB Chow [5].

Examining the top 25 genes ranked by fold change from this ‘RYGB-Specific HFD-Induced’ subset (Figure 5B) largely highlighted key downregulated transcriptional changes, as these sets were dominated by downregulated genes. These included *Gsta1*, *Gsta13*, and *Gstm3*, implicating altered glutathione and xenobiotic metabolism; *Hamp* and *Hamp2*, underscoring impaired iron regulation; and *Cyp2a4* and *Ephx1*, pointing to shifts in P450-based detoxification. While these gene-level observations align with the enrichment analysis findings for downregulated pathways, it is important to note that this heatmap does not capture the broader balance of upregulated and downregulated genes within the dataset. Altogether, these transcriptional changes illustrate how HFD continues to burden RYGB-operated mouse livers by upregulating inflammatory, coagulatory, and cholesterol-synthetic genes while hampering detoxification and iron balance.

Consistent with these gene-level signals, RYGB HFD mice displayed elevated fecal lipid content, lower plasma adiponectin, and increased liver glycogen relative to RYGB Chow, along with other tissue specific deficits such as greater bone mineral density loss and vitamin D deficiency, thereby showing that a HFD can still significantly constrain RYGB’s therapeutic scope [5]. Future research should clarify how these molecular pathways intersect to influence long-term NAFLD progression, iron deficiency, and lipid dysregulation in a RYGB context.

## 5. Discussion

This study provides a detailed analysis of how RYGB modulates the hepatic transcriptome, emphasizing post-operative dietary composition as a critical factor. Our approach diverges from many prior RYGB studies in mice, which often use only HF or “choice” diets, by including both chow and HFD groups. This design disentangles surgical effects from weight loss and obesity-driven influences, revealing pathways consistently altered by RYGB under both dietary conditions, as well as those uniquely affected by chow or HFD.

To begin with, our data show that although simple dietary normalization (Sham Chow) corrects many obesity-related phenotypes, RYGB imposes additional regulatory layers to achieve a more robust metabolic reorganization than diet alone [5,40,41]. Two major themes emerged from the upregulated transcriptomic pathways in RYGB-treated livers: “Extracellular space” factors that may remodel intercellular environments and iron handling, and genes labeled “adipogenesis genes,” which likely indicate more complex hepatic adaptations and possible liver-adipose crosstalk rather than straightforward adipocyte differentiation. For instance, *Trib3*, more recently linked to FGF21-driven lipid utilization under nutrient stress, surfaced in this “adipogenesis” cluster [42]. Interestingly, Trib3’s role in nutrient handling aligns with our observation of reduced fecal energy in these RYGB mice [5]. Collectively, these findings suggest that, while dietary normalization can resolve many obesity-related liver issues, RYGB engages distinct hepatic adaptations that enable coping with nutrient challenges, including iron deficiency, through a more nuanced regulatory network.

Alongside *Trib3*, other genes grouped within “Extracellular space” or “adipogenesis genes” pathways, such as *Socs3* and *Fgl1*, appear to have non-canonical (or underexplored) roles in iron regulation. Though “Iron Homeostasis” was not significantly enriched overall, several classical iron-regulatory genes were present in the RYGB-Induced gene set, including *Hamp*, *Hamp2*, *Bmp6*, *Id1* (all downregulated) and *Tfrc*, *Slc11a2*/*Dmt1*, and *Pzp* (all upregulated), with five of these genes appearing among the top 25 DEG’s (by fold-change/*p*-value), as shown in heatmap analyses. Taken together, these transcriptional changes indicate compensatory mechanisms that mitigate the local and systemic iron deficiency observed in these mice, though they are easily overlooked by standard pathway annotations.

**Figure 6:**
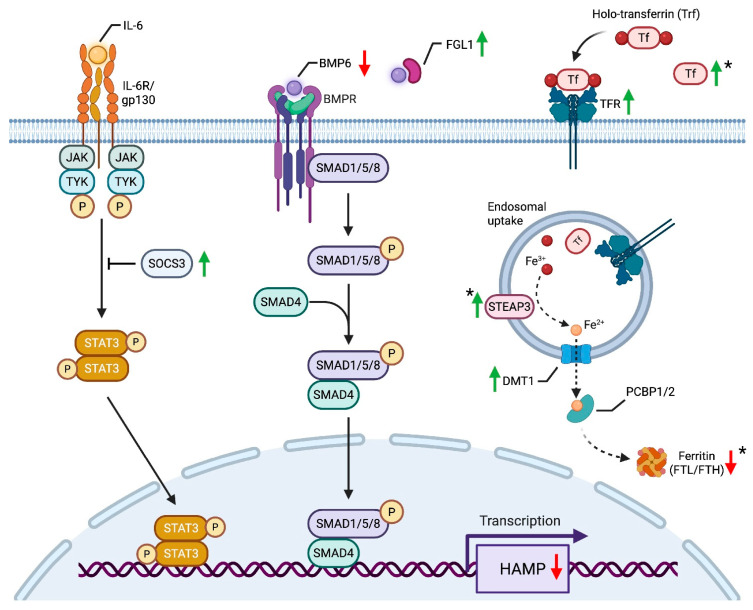
Proposed model of RYGB-induced hepatic transcriptional regulation of hepcidin and systemic iron homeostasis. This schematic presents a theoretical framework based on RNA-seq data, illustrating how RYGB-driven transcriptional changes may fine-tune hepcidin (Hamp) regulation to mitigate severe iron deficiency while preserving metabolic and inflammatory signaling. Two primary pathways involved in hepcidin regulation are highlighted: the IL-6-GP130-JAK/STAT3 and BMP-SMAD signaling axes. Increased *Socs3* expression following RYGB may attenuate IL-6-driven STAT3 phosphorylation by inhibiting JAK activity, potentially modulating IL-6’s metabolic effects without fully suppressing its downstream signaling. Similarly, upregulation of *Fgl1* suggests a possible antagonistic effect on BMP6, which could suppress BMP-SMAD signaling and further contribute to Hamp downregulation. Consistent with these transcriptional changes, RYGB may reduce hepatic iron sequestration while increasing systemic iron availability. Additional iron-handling genes exhibit altered expression, including upregulation of *Trf* and *Tfrc*, which could enhance iron uptake, and downregulation of ferritin storage proteins (*Ftl*/*Fth*), potentially limiting hepatic iron retention. Increased Steap3 and Dmt1 expression suggests enhanced mobilization and import of Fe^3+^/Fe^2+^ for cellular uptake. Genes specifically regulated under RYGB + HFD (*Ftl1*, *Fth1*, *Steap3*, *Trf*) are marked with an asterisk (*). Green arrows indicate upregulated genes, while red arrows indicate downregulated genes in response to RYGB.

When considering these underexplored iron-regulatory roles, it becomes clear that a prominent outcome of these transcriptional changes is the fine-tuning of hepcidin to avert severe iron deficiency while preserving beneficial inflammatory and metabolic signals. In particular, *Socs3* and *Fgl1*, identified in the upregulated enriched pathways, intersect with IL-6-GP130-JAK/STAT3 and BMP-SMAD, the two major axes driving hepcidin production [43]. Interestingly, SOCS3 suppresses JAK activity rather than directly inhibiting STAT3, suggesting that it moderates upstream signal amplification in the IL-6–JAK/STAT3 pathway [44,45]. This nuanced regulation may allow SOCS3 to fine-tune hepcidin production without completely abolishing STAT3 activation, thereby preserving IL-6’s beneficial metabolic effects, such as macrophage reprogramming and anti-inflammatory signaling [44,45]. Meanwhile, FGL1 antagonizes BMP6 [46], providing an additional check on hepcidin. Consistent with this, the downregulation of *Id1*, an established BMP6 target [47], further supports reduced BMP6 activity, reinforcing the notion that multiple factors contribute to hepcidin modulation post-RYGB. Notably, *Hif2α*, also elevated post-RYGB, can reduce BMP activity via EPO-ERFE production, further moderating hepcidin and helping preserve the IL-6–HIF2α synergy needed for protective macrophage reprogramming [48]. Through such orchestrated control, hepcidin regulation may prevent iron deficiency from becoming severe while allowing IL-6, regardless of its levels [49,50,51], to exert its favorable effects on metabolism and inflammation (Figure 6).

Downregulated pathways offer additional insight into the liver’s post-RYGB reconfiguration. Reduced expression of genes involved in mitochondrial function, such as *Acad9*, *Ndufb7*, *Ndufs5*, and iron-sulfur cluster proteins (*Alas2*, *Cmc1*), could signify diminished oxidative phosphorylation but may also be the results of altered iron availability. Concurrently, *ApoC3* (a major inhibitor of triglyceride clearance) was markedly suppressed, along with *ApoA2*, *ApoC1*, *ApoA5*, and *Angptl3*, pointing to broad remodeling of lipoprotein metabolism. Together with the upregulation of *Lsr*, which may enhance hepatic clearance of triglyceride-rich lipoproteins, these gene-expression shifts correlate well with the lower triglyceride levels observed clinically and in our mouse model of RYGB [5].

Taken together, these findings illustrate how RYGB reprograms the liver to balance nutrient stress, iron homeostasis, and immune signaling. By calibrating hepcidin through SOCS3-, FGL1-, and HIF2α-mediated control of IL-6-STAT3 and BMP-SMAD, RYGB mitigates the risk of profound iron deficiency while preserving beneficial macrophage and metabolic adaptations. Striking this balance is essential to support improved lipid handling, glucose control, and inflammation resolution in the post-RYGB state. These findings align with broader evidence that iron metabolism is tightly integrated with lipid homeostasis, influencing cholesterol biosynthesis, lipid oxidation, and metabolic inflammation [52]. Notably, while Rockfield et al. highlight how iron excess can drive lipid peroxidation and ferroptosis, our data suggest that the iron-deficient state post-RYGB may mitigate these processes, underscoring the dynamic interplay between iron levels and hepatic lipid metabolism.

Building on these RYGB-specific transcriptomic shifts, we identified a subset of “Reversal” genes that counter HF-induced changes. Though discussed in detail earlier, this set reinforces RYGB’s capacity to mitigate diet-driven hepatic dysregulation beyond standard dietary normalization. Noteworthy here is *Igfbp2*, already implicated in mediating RYGB’s systemic effects [53]. *Igfbp2* was robustly upregulated, potentially contributing to improved metabolic outcomes after surgery, whereas Igfbp5 showed an opposing downregulation (Supplementary Figure S7A,B). Although *Igfbp5* did not appear in the top 25 DEGs or enrichment overlap, its counter-regulation with *Igfbp2* highlights a possible therapeutic axis within the IGFBP family worthy of further investigation.

Of the eight “standout” genes identified in the Reversal subset, *Dvl1* and *Dglucy* stand out as the only two genes consistently ranking within the top 25 DEG’s across the RYGB-Induced, Reversal, and HFD-Induced gene sets. This shared prominence emphasizes their central role in the RYGB-mediated reversal of diet-induced obesity (DIO) dysregulation. *Dvl1*, a core component of the Wnt signaling pathway, has been studied in the context of obesity and NAFLD but remains unexplored in RYGB research. Its role in canonical Wnt signaling suggests it may contribute to liver reprogramming and metabolic improvements post-surgery. Similarly, *Dglucy*, a mitochondrial D-glutamate cyclase, supports glutamate homeostasis, a function important to mitochondrial energy dynamics and recently linked to DIO-induced mitochondrial dysfunction [54].

In addition to the eight standout genes, other individual targets such as Dact2 merit further study. *Dact2* showed high significance and, as a TGFBR suppressor, may counteract the fibrosis observed in obesity [55], even if TGF-β remains unchanged. Similarly, novel genes with high abundance but limited PubMed association, such as *Carhsp1* and *Abhd3*, emerge as intriguing candidates for further investigation. For instance, *Carhsp1*, regulated by nutrient status in the liver, inhibits gluconeogenic genes such as G6Pc and PEPCK1 by suppressing PPARα transcriptional activity [56]. *Abhd3*, a serine hydrolase involved in lipid metabolism, is part of a class of enzymes more recently proposed as therapeutic targets for lipid-related diseases due to its distinct role in medium-chain phospholipid catabolism [57]. These findings underscore how RYGB reprograms metabolic networks to restore signaling and mitochondrial function, revealing novel therapeutic targets for obesity-driven dysregulation.

While RYGB imposes a distinct transcriptomic program, high-fat feeding introduces a robust “diet signature” that persists across both surgical and sham groups. Pathway analyses highlighted translational repression, proteasome downregulation, and other changes consistent with histological findings of steatosis, lobular inflammation, and dyslipidemia in HF-fed mice. Downregulated ribosomal and proteasome genes hint at reduced translational efficiency and protein turnover, factors that can compromise hepatic proteostasis. Meanwhile, continued activation of inflammatory cascades underscores a persistent pro-inflammatory milieu conducive to hepatic injury.

Nevertheless, these HFD-driven changes remain partially operative in RYGB mice, underscoring that dietary fat exerts a potent hepatic influence independent of body weight. Indeed, many genes commonly labeled “obesity-related” are more accurately described as “HFD-induced,” highlighting the need to disentangle dietary effects from overall adiposity. By identifying genes that respond robustly to HFD regardless of surgical status, our findings clarify how dietary lipids shape hepatic transcriptional programs even in the face of substantial weight loss.

Building on the baseline “diet signature,” the RYGB-Specific HFD-Induced transcriptome reveals additional layers of metabolic dysregulation driven by HF feeding, including amplified iron dysregulation and oxidative stress. Classically annotated pathways, such as “coagulation,” “cholesterol biosynthesis,” and “xenobiotic metabolism,” unexpectedly converge with genes regulating iron handling, oxidative stress, and fibrosis. *Hamp*, *Hamp2*, *Pzp*, and *Fgl1*, previously identified in the RYGB-Induced gene set, remain prominent and are further intensified, while new players such as *Ftl1*, *Fth1*, *Trf*, and *Steap3* emerge, signaling exacerbated iron dysregulation under HFD.

As mentioned previously, “Iron Homeostasis” was not enriched in the RYGB-Induced set, likely due to novel contributions from non-canonical regulators like *Fgl1* and *Socs3*. Under HFD stress, however, significant enrichment emerges, driven by genes such as *Hamp*, *Hamp2*, *Ftl1*, and *Fth1*, further supporting the idea that HFD amplifies iron dysregulation. Although Hmox1 was not upregulated, several heme-scavenging genes (*Hpx*, *Lrp1*/*Cd91*) were induced, while others involved in heme or biliverdin handling (*Slc48a1*/*Hrg1*, *Pgrmc1*, *Blvrb*) were downregulated, suggesting a compensatory shift in heme-related processes consistent with iron deficiency. Additional changes, including the downregulation of *Gpx4* and xenobiotic detoxification genes (*Ephx1*, *Gsta1*, *Gstm3*), suggest a reprioritization of hepatic resources toward critical processes like systemic iron homeostasis under oxidative stress. Together, these transcriptional shifts may reveal how the post-RYGB liver contends with heightened lipid influx while prioritizing systemic iron homeostasis, often at the expense of oxidative stress resilience and detox capacity.

These patterns do not occur under chow-fed or sham-operated conditions, highlighting the novel convergence of cholesterol biosynthesis, xenobiotic detoxification, and iron-regulatory pathways under HFD stress. Genes such as *Scarb1* and *Fn1* illustrate the pleiotropy of these responses: *Scarb1*, beyond its established role in HDL uptake and cholesterol trafficking [58], also suppresses ferroptosis and modulates inflammation [59,60,61]. Similarly, *Fn1* (fibronectin), traditionally linked to extracellular matrix organization, also emerges in coagulation pathways, suggesting it may contribute to clot stabilization and fibrosis during liver injury. *Lrg1* contributes to acute-phase responses and TGF-β–mediated fibrosis [62], amplifying hepatic vulnerability under RYGB and HFD-induced metabolic stress. Collectively, these findings reveal how “lipid-centric” or “acute-phase” pathways adapt to meet the challenges of the post-RYGB liver but remain insufficient to fully resolve fibrosis and metabolic dysfunction. Relatively speaking, however, even though the HFD-induced burden far outweighs the smaller set of RYGB-HFD genes, that smaller cohort still exerts a sufficiently strong compensatory effect to yield a net improvement over Sham HFD, underscoring how RYGB helps restrain the degree of liver pathology typically seen under HF feeding.

Future work might explore targeted interventions to address specific vulnerabilities identified in this study, such as optimizing dietary iron to mitigate systemic deficiency, modulating glutathione-S-transferases to enhance resilience against oxidative stress, or exploring selective inhibitors of hepatic cholesterol biosynthesis to counteract the lingering dyslipidemia under RYGB + HFD conditions. By addressing these interconnected pathways, we may better preserve RYGB’s benefits and reduce NAFLD relapse under HF feeding. Ultimately, these findings underscore that dietary composition amplifies the hepatic transcriptomic reprogramming initiated by RYGB. By addressing the unique vulnerabilities introduced by HF feeding, such as dysregulated iron homeostasis and impaired oxidative resilience, we may optimize post-RYGB liver health and further enhance the surgery’s metabolic benefits.

## 6. Conclusions

These findings highlight the liver’s pivotal role in mediating RYGB’s metabolic benefits, as well as the complex interplay of surgical and dietary influences. By distinguishing “RYGB-Induced,” “HFD-Induced,” and “RYGB-Specific HFD-Induced” genes, we show that RYGB confers resilience against obesogenic stress but cannot fully neutralize the adverse effects of high fat. Tailored post-operative diets, especially reducing dietary fat, may be crucial for protecting long-term liver health and preventing partial NAFLD relapse. It is important to note that we focused on fasted-state transcriptomes, which may overlook dynamic, meal-driven responses. Moreover, while we lack direct protein-level validation here, these observations are consistent with our previous study on the same mice, which included comprehensive analyses of iron regulatory genes at both mRNA and protein levels [5]. Future research could employ proteomic or metabolomic approaches to capture real-time responses missed by fasted-state RNA-seq.

Overall, this work explores RYGB’s power to recalibrate hepatic function while also revealing how HF intake can diminish these gains. By mapping the liver’s transcriptional landscape, we identify molecular levers that could enhance RYGB’s benefits or inform novel therapies. In sum, hepatic adaptation after RYGB represents a balance between surgical reprogramming and dietary pressures. A deeper understanding of these interactions, grounded in multi-omic validations, will help optimize dietary recommendations and potential adjunct therapies, ultimately improving long-term outcomes in RYGB-treated, bariatric populations.

## Data Availability

The raw data supporting the conclusions of this article will be made available by the authors on request.

## Supplementary Materials

The following supporting information can be downloaded at: https://www.mdpi.com/article/10.3390/jcm15020479/s1, Figure S1: Data preprocessing and filtering for RNA-seq analysis. (A) Bar plot of total raw read counts (millions) for each sample across experimental groups. (B) Histogram of SNR values across genes, with inflection points at SNR = 1.9 (blue) and SNR = 4.1 (red). (C) Scatterplot showing the relationship between SNR and mean counts per million (CPM). Dotted lines indicate where the SNR inflection points identified in panel (B) correspond to mean CPM thresholds (CPM = 0.59 for SNR = 1.9 and CPM = 5.1 for SNR = 4.1), helping establish filtering criteria. (D) Histogram of log2-transformed CPM values, highlighting inflection (1.5) and trough (2.3) points translating to CPM values of 1.82 and 3.92, respectively. (E) Density plot showing expression level distributions across experimental groups following CPM-based filtering. Figure S2: Body weight discrepancies and their impact on retention of significant genes after FDR correction. (A) Boxplots showing significantly higher body weights in Sham HFD mice compared to other groups, introducing a confounding variable. (B) Percentage of differentially expressed genes retained after FDR correction, with contrasts involving Sham HFD retaining disproportionately more significant genes, reflecting inflated statistical power due to body weight discrepancies. (C) Total number of significant genes retained post-FDR correction, highlighting the confounding effects of body weight differences and the risks of excluding biologically validated changes with stringent thresholds. Figure S3: Identification of commonly regulated gene sets across experimental conditions. (A) Venn diagram illustrating RYGB-induced effects, defined as genes that were significantly and consistently regulated in the same direction in both Sham Chow vs. RYGB Chow and Sham HFD vs. RYGB HFD comparisons (365 genes; Common Regulation). (B) Venn diagram depicting Reversal genes, a subset of RYGB-induced genes that were regulated in the opposite direction compared to Sham Chow vs. Sham HFD, indicating RYGB-driven reversal of obesity-associated transcriptional changes (119 genes; Inverse Regulation). (C) Venn diagram showing HFD-induced effects, defined as genes that were significantly and consistently regulated in the same direction in both Sham Chow vs. Sham HFD and RYGB Chow vs. RYGB HFD, identifying transcriptional changes robustly driven by HFD exposure (860 genes; Common Regulation). (D) Venn diagram illustrating RYGB-specific HFD effects, derived by removing HFD-induced genes from the RYGB Chow vs. RYGB HFD contrast. This subset highlights transcriptional changes unique to the interaction between RYGB and HFD (426 genes; Common Regulation). These gene sets (A–D) formed the basis for pathway enrichment and further transcriptional analyses in this study. Figure S4: Heatmaps of transcriptional changes for complete gene sets. Heatmaps of scaled rlog-transformed expression values for all genes in the (A) RYGB-Induced (365 genes), (B) Reversal (119 genes), (C) HFD-Induced (860 genes), and (D) RYGB-Specific HFD-Induced (426 genes) sets, illustrating transcriptional changes across experimental groups. Figure S5: Permutation analysis validating the overlap of commonly significant genes. Histograms showing the distribution of overlapping significant genes across 10,000 random permutations for (A) RYGB-induced comparisons and (B) HFD-induced comparisons. The observed overlaps (red dashed lines) are significantly greater than expected by chance (*p* < 0.0002 and *p* < 0.0001, respectively). Figure S6: PubMed associations for RYGB-induced and related gene sets. Scatterplots of average gene abundance (log10) versus PubMed hits for keyword categories. Categories include (A) Obesity, (B) RYGB, (C) Liver, (D) NAFLD, (E) Iron, and (F) an aggregate metric, highlighting genes with high abundance and literature representation across all categories. Figure S7: Expression dynamics of Igfbp2 and Igfbp5 across experimental groups. Boxplots of normalized expression counts for (A) Igfbp2, showing significantly higher expression in RYGB groups compared to Sham, and (B) Igfbp5, showing reduced expression in RYGB groups, suggesting counter-regulatory dynamics with Igfbp2.
