# Supplementary material for: Analysis of Roux-en-Y Gastric Bypass and High-Fat Feeding Reveals Hepatic Transcriptome Reprogramming: Ironing out the Details
Source: J Clin Med. 2026 Jan 7;15(2):479. doi: 10.3390/jcm15020479 (PMC12841844; doi:10.3390/jcm15020479)
Supplement: Supplementary file 1 [file jcm-15-00479-s001.zip › jcm-4054687-supplementary.pdf]

Supplementary Figures

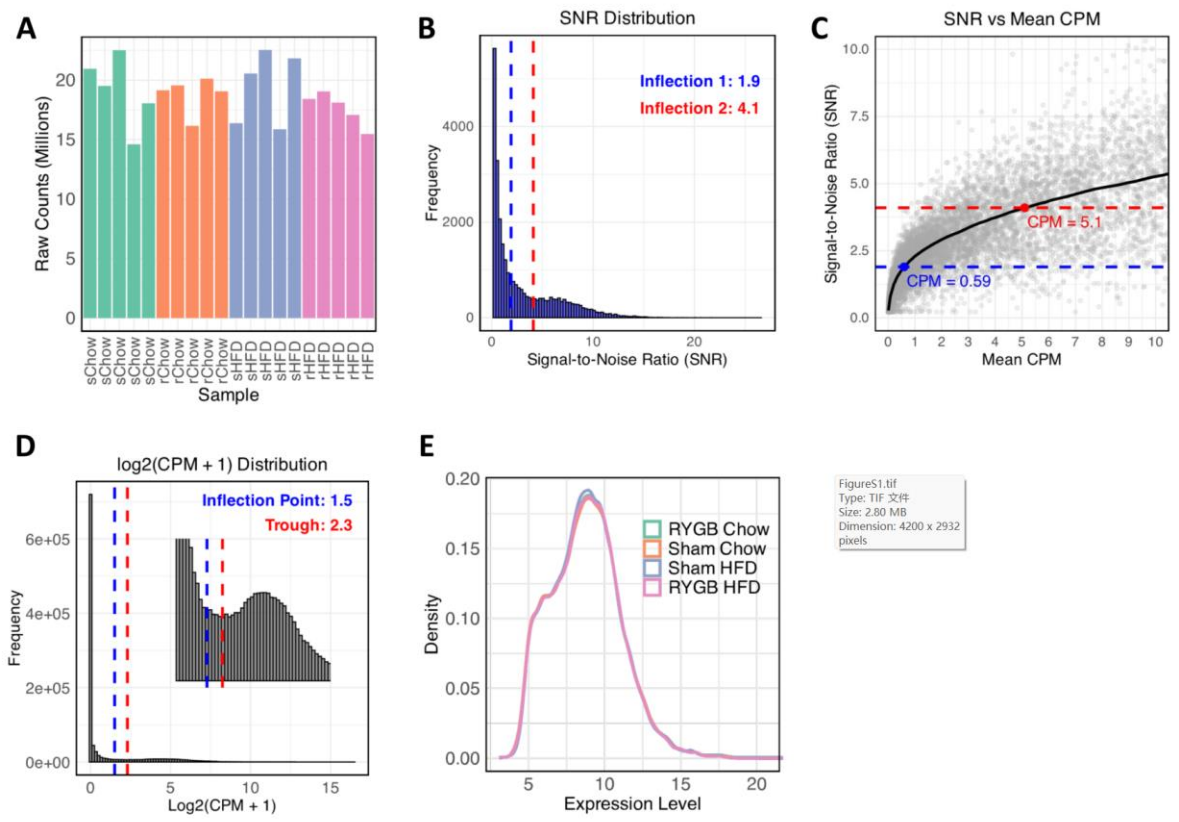

Supplementary Figure S1. Data preprocessing and filtering for RNA-seq analysis. (A) Bar plot of total raw read counts (millions) for each sample across experimental groups. (B) Histogram of SNR values across genes, with inflection points at SNR = 1.9 (blue) and SNR = 4.1 (red). (C) Scatterplot showing the relationship between SNR and mean counts per million (CPM). Dotted lines indicate where the SNR inflection points identified in panel (B) correspond to mean CPM thresholds (CPM = 0.59 for SNR = 1.9 and CPM = 5.1 for SNR = 4.1), helping establish filtering criteria. (D) Histogram of log2-transformed CPM values, highlighting inflection (1.5) and trough (2.3) points translating to CPM values of 1.82 and 3.92, respectively. (E) Density plot showing expression level distributions across experimental groups following CPM-based filtering.

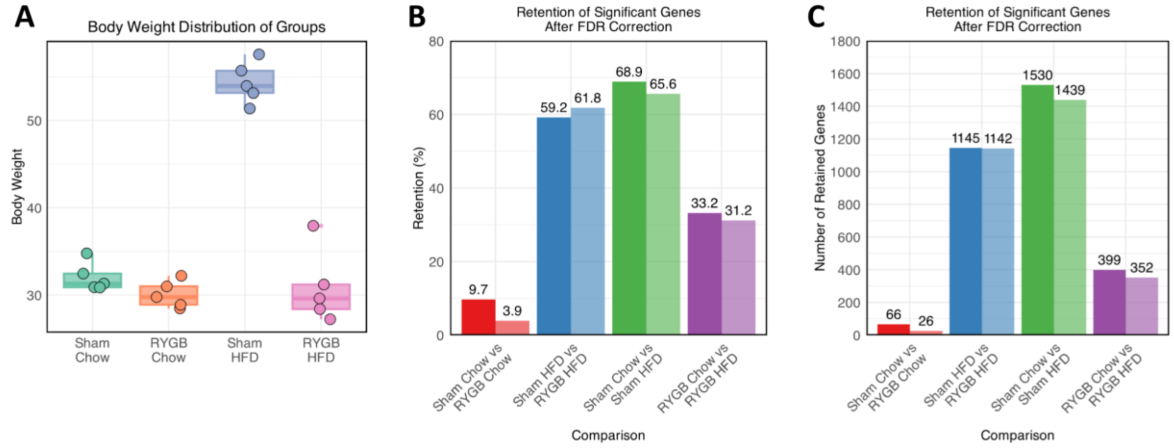

Supplementary Figure S2. Body weight discrepancies and their impact on retention of significant genes after FDR correction. (A) Boxplots showing significantly higher body weights in Sham HFD mice compared to other groups, introducing a confounding variable. (B) Percentage of differentially expressed genes retained after FDR

correction, with contrasts involving Sham HFD retaining disproportionately more significant genes, reflecting inflated statistical power due to body weight discrepancies. (C) Total number of significant genes retained post-FDR correction, highlighting the confounding effects of body weight differences and the risks of excluding biologically validated changes with stringent thresholds.

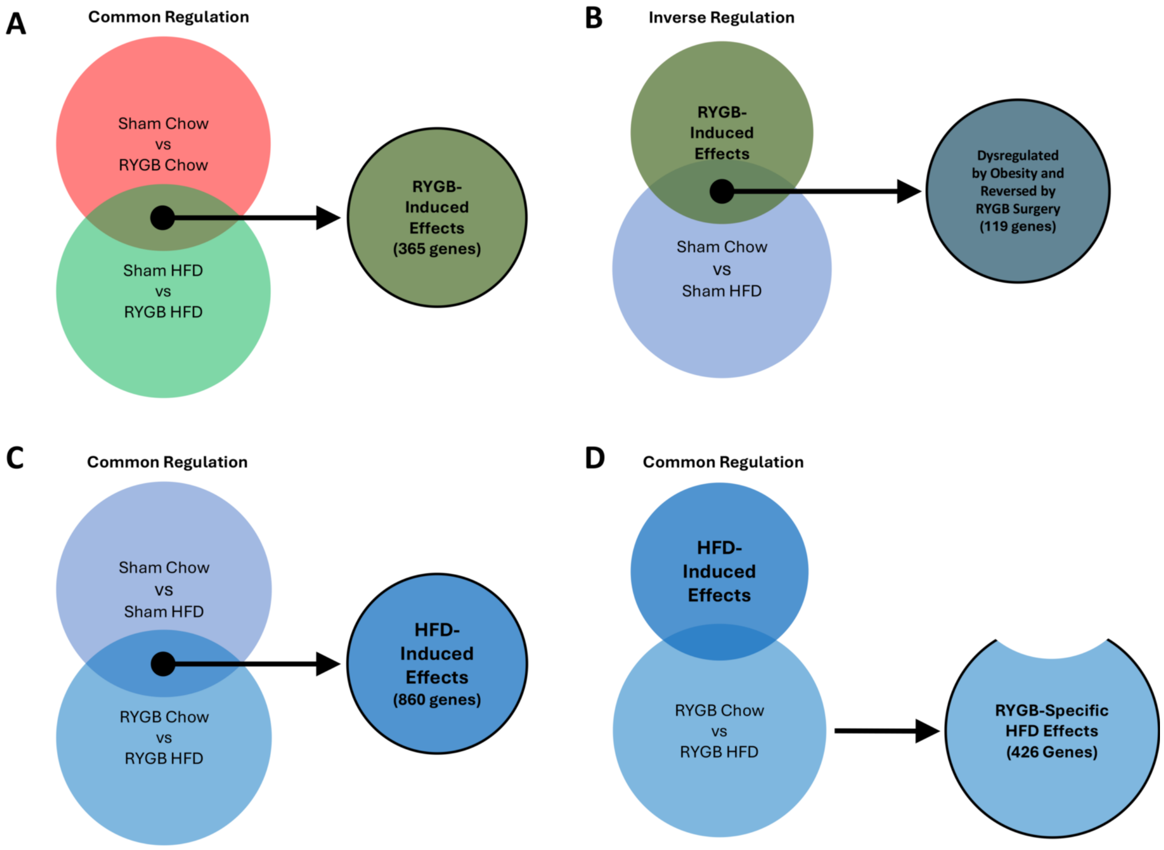

Supplementary Figure S3. Identification of commonly regulated gene sets across experimental conditions. (A) Venn diagram illustrating RYGB-induced effects, defined as genes that were significantly and consistently regulated in the same direction in both Sham Chow vs. RYGB Chow and Sham HFD vs. RYGB HFD comparisons (365 genes; Common Regulation). (B) Venn diagram depicting Reversal genes, a subset of RYGB-induced genes that were regulated in the opposite direction compared to Sham Chow vs. Sham HFD, indicating RYGB-driven reversal of obesity-associated transcriptional changes (119 genes; Inverse Regulation). (C) Venn diagram showing HFD-induced effects, defined as genes that were significantly and consistently regulated in the same direction in both Sham Chow vs. Sham HFD and RYGB Chow vs. RYGB HFD, identifying transcriptional changes robustly driven by HFD exposure (860 genes; Common Regulation). (D) Venn diagram illustrating RYGB-specific HFD effects, derived by removing HFD-induced genes from the RYGB Chow vs. RYGB HFD contrast. This subset highlights transcriptional changes unique to the interaction between RYGB and HFD (426 genes; Common Regulation). These gene sets (A-D) formed the basis for pathway enrichment and further transcriptional analyses in this study.

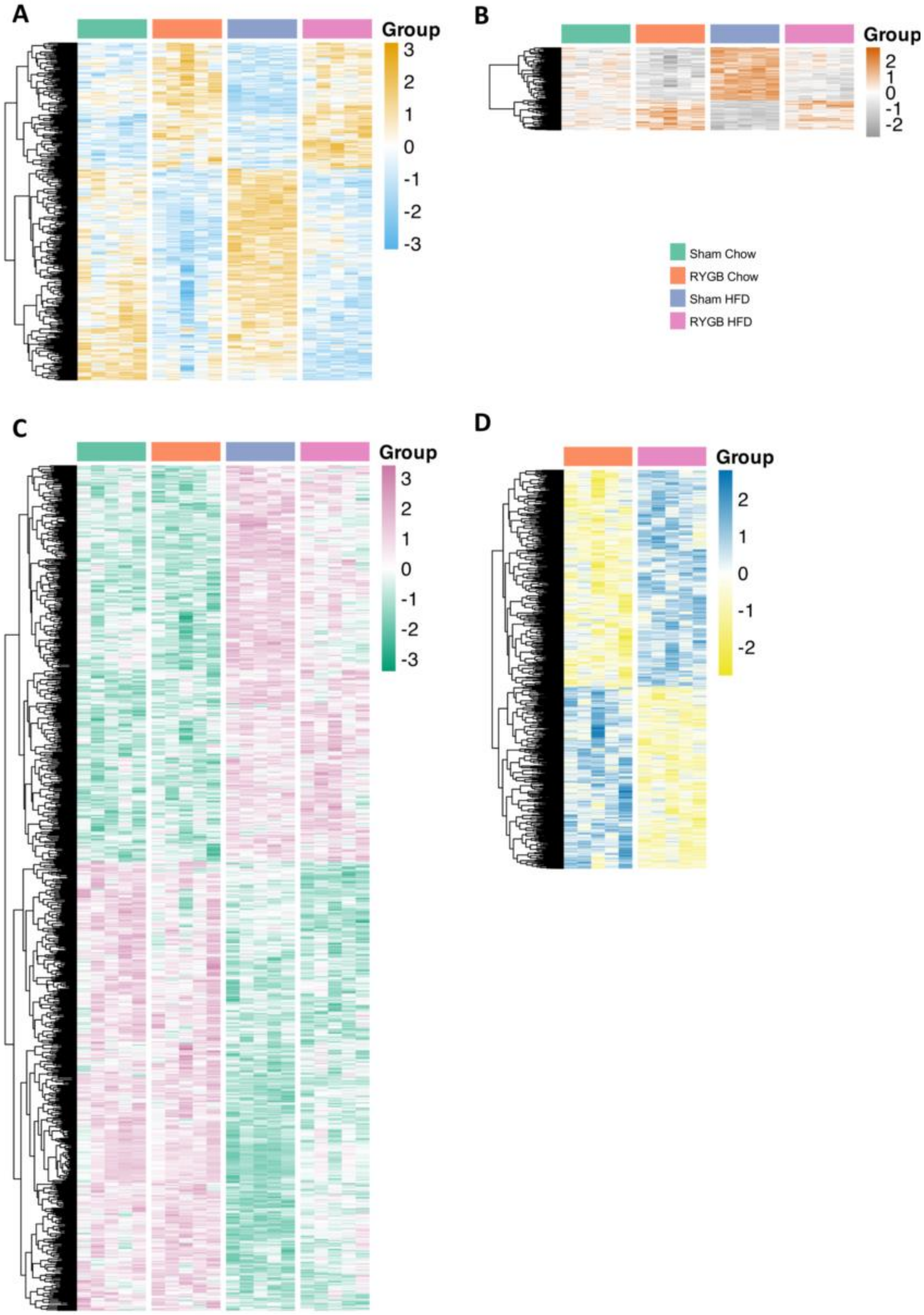

Supplementary Figure S4. Heatmaps of transcriptional changes for complete gene sets. Heatmaps of scaled rlog-transformed expression values for all genes in the (A) RYGB-Induced (365 genes), (B) Reversal (119 genes),

(C) HFD-Induced (860 genes), and (D) RYGB-Specific HFD-Induced (426 genes) sets, illustrating transcriptional changes across experimental groups.

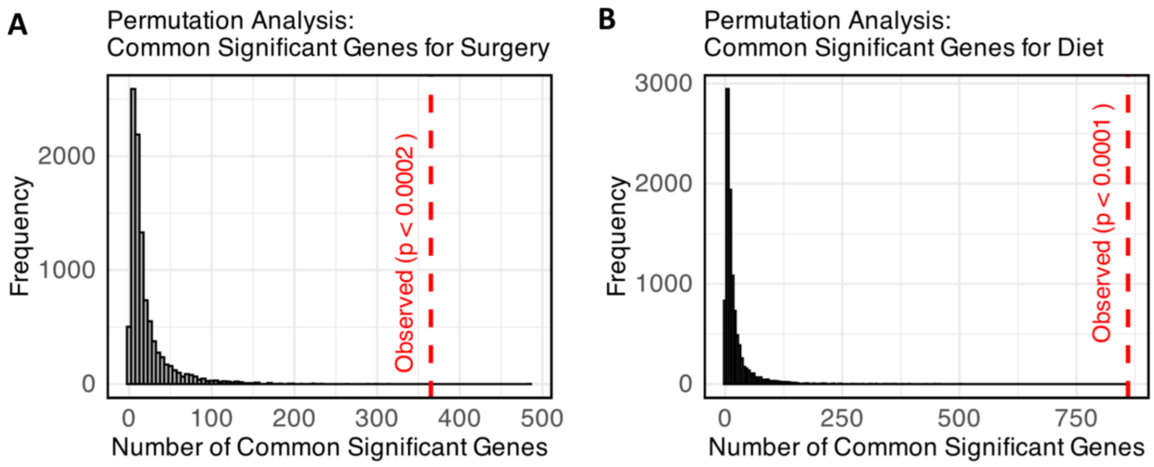

Supplementary Figure S5. Permutation analysis validating the overlap of commonly significant genes. Histograms showing the distribution of overlapping significant genes across 10,000 random permutations for (A) RYGB-induced comparisons and (B) HFD-induced comparisons. The observed overlaps (red dashed lines) are significantly greater than expected by chance ($p < 0.0002$ and $p < 0.0001$, respectively).

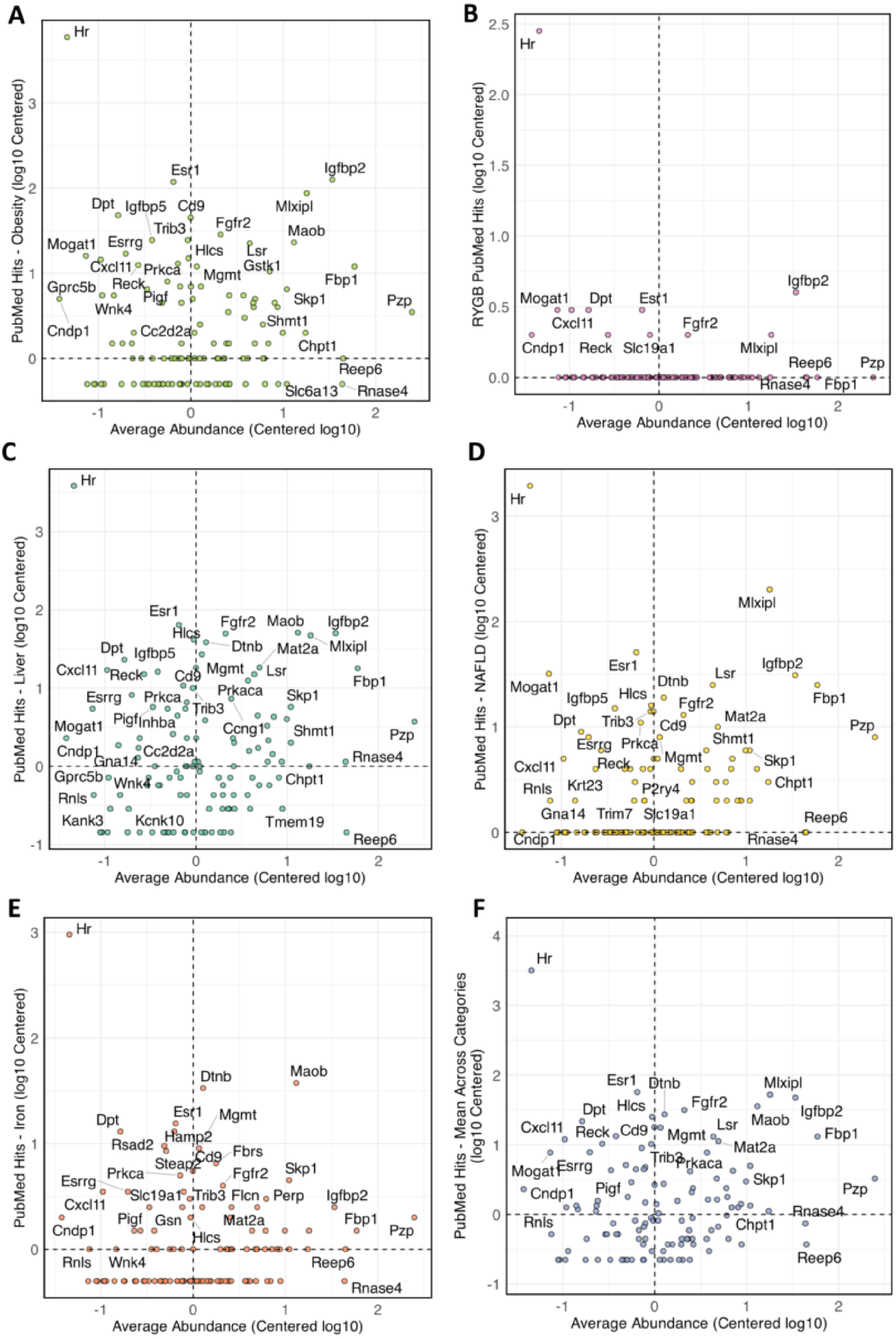

A
PubMed Hits - Obesity (log10 Centered)
Average Abundance (Centered log10)
Hr
Esr1
Igfbp2
Dpt
Igfbp5
Cd9
Mlxipl
Trib3
Fgfr2
Maob
Mogat1
Esrrg
Hlcs
Lsr
Cxcl11
Prkca
Mgmt
Gstk1
Reck
Fbp1
Gprc5b
Pigf
Skp1
Pzp
Wnk4
Shmt1
Cndp1
Cc2d2a
Chpt1
Reep6
Slc6a13
Rnase4
B
RYGB PubMed Hits (log10 Centered)
Average Abundance (Centered log10)
Hr
Igfbp2
Mogat1
Dpt
Esr1
Cxcl11
Fgfr2
Cndp1
Reck
Slc19a1
Mlxipl
Reep6
Pzp
Rnase4
Fbp1
C
PubMed Hits - Liver (log10 Centered)
Average Abundance (Centered log10)
Hr
Esr1
Fgfr2
Maob
Igfbp2
Hlcs
Dtnb
Dpt
Igfbp5
Mat2a
Mlxipl
Cxcl11
Reck
Cd9
Mgmt
Lsr
Fbp1
Prkaca
Esrrg
Prkca
Skp1
Trib3
Pigf
Inhba
Mogat1
Ccng1
Shmt1
Pzp
Cndp1
Cc2d2a
Rnase4
Gna14
Gprc5b
Wnk4
Chpt1
Rnls
Kank3
Kcnk10
Tmem19
Reep6
D
PubMed Hits - NAFLD (log10 Centered)
Average Abundance (Centered log10)
Hr
Mlxipl
Esr1
Igfbp2
Dtnb
Lsr
Mogat1
Igfbp5
Hlcs
Fbp1
Fgfr2
Dpt
Trib3
Cd9
Mat2a
Pzp
Esrrg
Prkca
Shmt1
Reck
Mgmt
Skp1
Cxcl11
Rnls
Krt23
P2ry4
Chpt1
Gna14
Trim7
Slc19a1
Reep6
Cndp1
Rnase4
E
PubMed Hits - Iron (log10 Centered)
Average Abundance (Centered log10)
Hr
Dtnb
Maob
Esr1
Mgmt
Dpt
Hamp2
Rsad2
Fbrs
Cd9
Steap2
Skp1
Prkca
Fgfr2
Esrrg
Slc19a1
Trib3
Flcn
Perp
Igfbp2
Cxcl11
Pigf
Gsn
Mat2a
Fbp1
Cndp1
Hlcs
Pzp
Rnls
Wnk4
Reep6
Rnase4
F
PubMed Hits - Mean Across Categories (log10 Centered)
Average Abundance (Centered log10)
Hr
Esr1
Dtnb
Mlxipl
Dpt
Hlcs
Fgfr2
Igfbp2
Maob
Lsr
Cxcl11
Reck
Cd9
Mgmt
Fbp1
Mat2a
Trib3
Prkaca
Mogat1
Esrrg
Skp1
Pigf
Pzp
Cndp1
Rnase4
Rnls
Chpt1
Reep6

Supplementary Figure S6. PubMed associations for RYGB-induced and related gene sets. Scatterplots of average gene abundance (log10) versus PubMed hits for keyword categories. Categories include (A) Obesity, (B) RYGB, (C) Liver, (D) NAFLD, (E) Iron, and (F) an aggregate metric, highlighting genes with high abundance and literature representation across all categories.

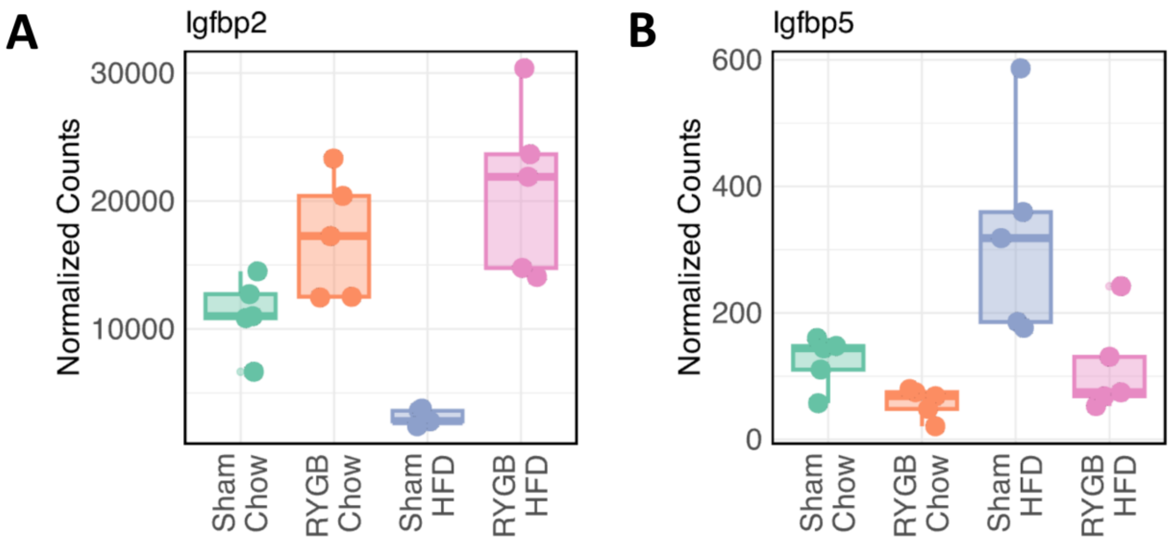

Supplementary Figure S7. Expression dynamics of Igfbp2 and Igfbp5 across experimental groups. Boxplots of normalized expression counts for (A) Igfbp2, showing significantly higher expression in RYGB groups compared to Sham, and (B) Igfbp5, showing reduced expression in RYGB groups, suggesting counter-regulatory dynamics with Igfbp2.
